# Acute undifferentiated fever in India: a multicentre study of aetiology and diagnostic accuracy

**DOI:** 10.1186/s12879-017-2764-3

**Published:** 2017-10-04

**Authors:** Kristine Mørch, Anand Manoharan, Sara Chandy, Novin Chacko, Gerardo Alvarez-Uria, Suvarna Patil, Anil Henry, Joel Nesaraj, Cijoy Kuriakose, Ashita Singh, Siby Kurian, Christel Gill Haanshuus, Nina Langeland, Bjørn Blomberg, George Vasanthan Antony, Dilip Mathai

**Affiliations:** 1 National Centre for Tropical Infectious Diseases, Department of Medicine, Haukeland University Hospital, Bergen, Norway; 2 Department of Clinical Science, University of Bergen, Bergen, Norway; 3 Infectious Diseases Training and Research Centre, Department of Medicine, Christian Medical College, Vellore, India; 4 Duncan Hospital, Raxaul, Bihar India; 5 Rural Development Trust Hospital, Anantapur, Andhra Pradesh India; 6 B.K.L. Walawalkar Hospital, Ratnagiri, Maharashtra India; 7 Christian Hospital, Mungeli, Chhattisgarh India; 8 Bethesda Hospital, Ambur, Tamil Nadu India; 9 Christian Fellowship Hospital, Oddanchatram, Tamil Nadu India; 10 Baptist Christian Hospital, Tezpur, Assam India

**Keywords:** Malaria, Bacteraemia, Leptospirosis, Scrub typhus, Dengue, Chikungunya, Prevalence, India, Diagnosis

## Abstract

**Background:**

The objectives of this study were to determine the proportion of malaria, bacteraemia, scrub typhus, leptospirosis, chikungunya and dengue among hospitalized patients with acute undifferentiated fever in India, and to describe the performance of standard diagnostic methods.

**Methods:**

During April 2011–November 2012, 1564 patients aged ≥5 years with febrile illness for 2–14 days were consecutively included in an observational study at seven community hospitals in six states in India.

Malaria microscopy, blood culture, Dengue rapid NS1 antigen and IgM Combo test, *Leptospira* IgM ELISA, Scrub typhus IgM ELISA and *Chikungunya* IgM ELISA were routinely performed at the hospitals.

Second line testing, Dengue IgM capture ELISA (MAC-ELISA), Scrub typhus immunofluorescence (IFA), *Leptospira* Microscopic Agglutination Test (MAT), malaria PCR and malaria immunochromatographic rapid diagnostic test (RDT) Parahit Total™ were performed at the coordinating centre. Convalescence samples were not available.

Case definitions were as follows: Leptospirosis: Positive ELISA and positive MAT. Scrub typhus: Positive ELISA and positive IFA. Dengue: Positive RDT and/or positive MAC-ELISA. Chikungunya: Positive ELISA. Bacteraemia: Growth in blood culture excluding those defined as contaminants. Malaria: Positive genus-specific PCR.

**Results:**

Malaria was diagnosed in 17% (268/1564) and among these 54% had *P. falciparum.* Dengue was diagnosed in 16% (244/1564). Bacteraemia was found in 8% (124/1564), and among these *Salmonella typhi* or *S. paratyphi* constituted 35%. Scrub typhus was diagnosed in 10%, leptospirosis in 7% and chikungunya in 6%. Fulfilling more than one case definition was common, most frequent in chikungunya where 26% (25/98) also had positive dengue test.

**Conclusions:**

Malaria and dengue were the most common causes of fever in this study. A high overlap between case definitions probably reflects high prevalence of prior infections, cross reactivity and subclinical infections, rather than high prevalence of coinfections. Low accuracy of routine diagnostic tests should be taken into consideration when approaching the patient with acute undifferentiated fever in India.

## Background

Infectious diseases are the leading causes of morbidity and death in India [1]. Field studies on fever aetiology in India are few, and surveillance is limited by lack of accessibility to health facilities. The wide uncertainty range is illustrated by the gap between approximately 1000 malaria deaths per year reported annually from India and estimated numbers between 20,000 and 200,000 per year [2–4]. In acute undifferentiated fever (AUF), symptoms are unspecific, and if accurate diagnostic methods are not available, empirical treatment needs to be broad in order to avoid deaths. Prevalence data and access to affordable, sensitive and specific diagnostic methods are tools to provide targeted and effective treatment of severe acute infections, and to avoid further development of antimicrobial resistance in India [5]. However, multiple positive diagnostic test results in the same patient are common, as shown by D’Acremont et al. in a study in Tanzania [6]. Positive tests due to subclinical or previous infections and cross reactivity in serological tests, makes interpretation of results a challenge. Awareness of the limitations and strengths of diagnostic tests is necessary both in the interpretation of epidemiological surveys and when approaching the individual fever patient.

The main objective of this study was to determine the proportion of AUF caused by malaria, bacteraemia, scrub typhus, leptospirosis, chikungunya and dengue among patients admitted to community hospitals in India. A secondary objective was to describe the performance of routine diagnostic methods.

## Methods

### Study sites and participants

**Fig. 1:**
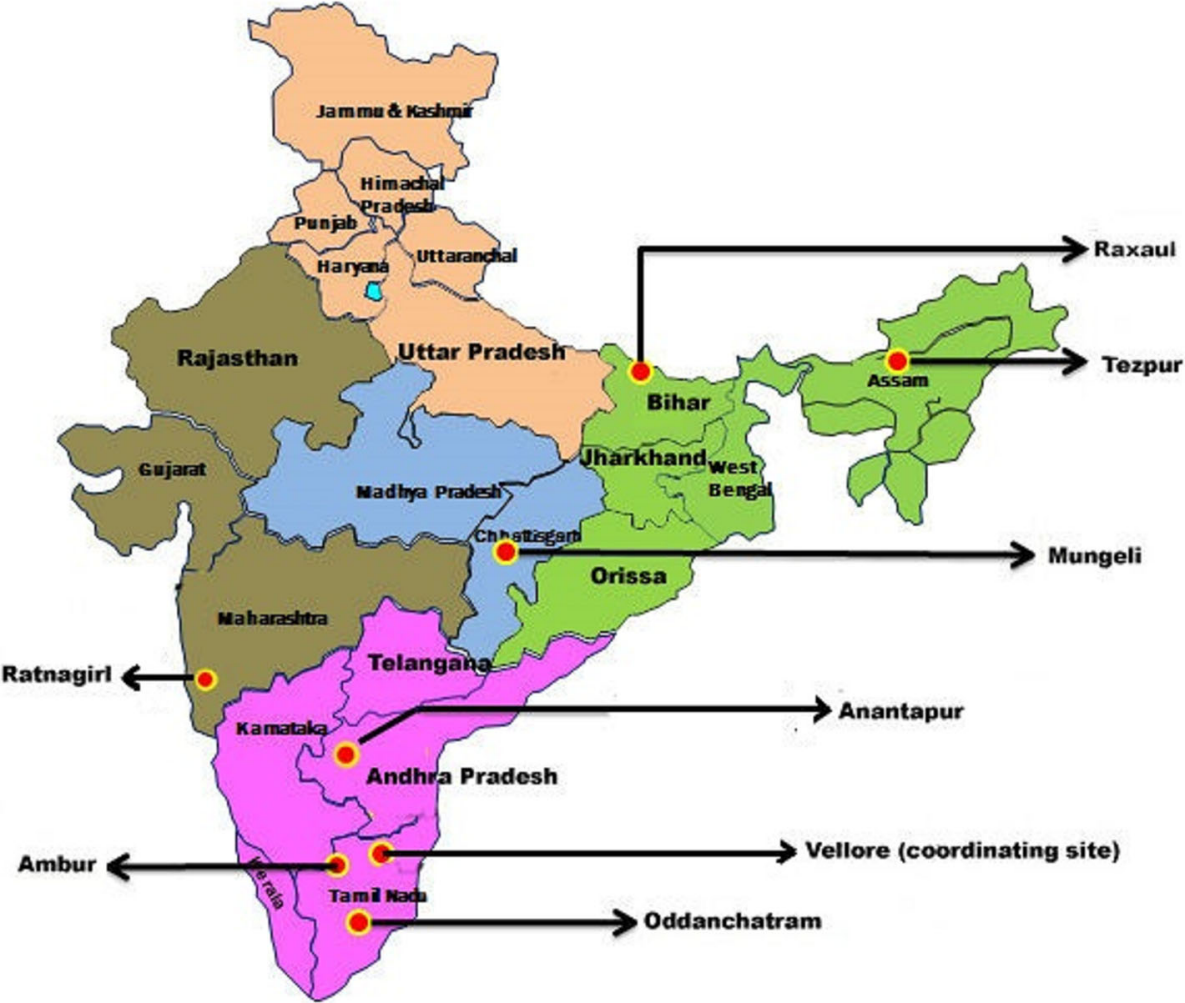
Location of hospitals in six states of India participating in the study

During April 2011–November 2012, patients aged ≥5 years admitted with AUF were consecutively included from the following secondary, community (100 to 500) bed hospitals: Baptist Christian Hospital in Tezpur (Assam, North East India), Duncan Hospital in Raxaul (Bihar, North India), Christian Hospital in Mungeli (Chhattisgarh, Central India), B.K. Walawalkar Hospital in Ratnagiri (Maharashtra, Western India), Rural Development Trust Hospital in Anantapur (Andhra Pradesh, South India), Christian Fellowship Hospital in Oddanchatram (Tamil Nadu, South India) and Bethesda Hospital in Ambur (Tamil Nadu, South India) (Fig. 1).

Details of the climate variation among the study sites have been published previously [7]. The study coordinating centre was Christian Medical College (CMC), Vellore, India.

AUF was defined as measured temperature ≥ 38 °C and history of febrile illness of 2–14 days duration, with no localized cause as judged by the treating physician. Patients were not excluded if they had abdominal pain, diarrhoea, haematochezia, nausea or vomiting, rhinorrhoea, dyspnoea, ocular pain, altered sensorium, headache, stiff neck, rash, arthralgia, myalgia, petechiae, ecchymosis, epistaxis, gingival bleeding or jaundice.

### Study procedures

#### Microbiological investigations

The following laboratory tests were performed at the study hospitals as part of routine investigation: Malaria blood smears, Scrub typhus IgM ELISA (In Bios, USA), *Leptospira* IgM ELISA (Panbio Pty., Ltd., Queensland, Australia), Chikungunya IgM ELISA (NIV, India), Dengue rapid NS1 antigen and IgM/IgG Combo test (SD bioline, USA) and blood cultures. Convalescence serology testing was not performed due to logistic challenges. In order to improve detection of IgM antibodies serological testing was delayed until five days of fever, if possible.

Blood was cultured with conventional methods, or automated (BACTEC, Becton Dickinson, Maryland, USA), and if growth was detected, the isolate was identified at each site and frozen, then in Transport swab (Hi Media, Mumbai, India) sent to the reference laboratory for re-identification and confirmation.

The following investigations were performed at the reference laboratory at CMC: Scrub typhus IgM ELISA (cut off value of 0.5 OD), *Leptospira* IgM ELISA, Chikungunya IgM ELISA and Dengue NS1/IgM Combo test, only if not performed at local site. Dengue IgM capture ELISA (MAC-ELISA) was performed at reference laboratory on all samples. Scrub typhus immunofluorescence (IFA) was performed on all IgM ELISA positives and some ELISA negatives. *Leptospira* Microscopic Agglutination Test (MAT) was performed on all IgM ELISA positives and some ELISA negatives.

The immunochromatographic malaria rapid diagnostic test (RDT) Parahit Total™ (Span Diagnostics Ltd., Surat, India) was performed on all samples. A *Plasmodium* genus-specific PCR targeting mitochondrial genome [8] was performed on all samples, and a species-specific PCR targeting 18S or sequencing was performed to identify species on those that were genus PCR positive. Details on the malaria diagnostic methods and results in this study have been reported previously [7].

Case definitions were as follows:

Leptospirosis: Positive ELISA and positive MAT.

Scrub typhus: Positive ELISA and positive IFA.

Dengue: Positive RDT and/or positive MAC-ELISA.

Chikungunya: Positive ELISA.

Bacteraemia: Growth of bacteria not considered to be contaminants in blood culture.

Malaria: Positive malaria genus-specific PCR.

All tests were performed as per the standard protocol provided by the manufacturers. Some serology tests were performed as a quality control with the same method both at the local centre and at the reference laboratory. In these cases a positive result was defined as two positive results or one positive and one equivocal, a negative result defined as two negatives or one negative and one equivocal, while one positive and one negative result was defined as discrepant.

### Statistical analysis

Chi-square test was used to assess differences between proportions.

## Results

A total of 1564 patients were included, with mean (median, range) age 34 (31, 5–105) years. Among these 632 (40%) were women and 895 (57%) were men, and 1219 (78%) lived in rural areas. Table 1 shows demographic characteristics for each study site.

**Table 1:** Demographic characteristics. *N* = 1564

Characteristics	Total	Oddanchatram	Ambur	Tezpur	Mungeli	Anantapur	Ratnagiri	Raxaul
Patients N (%)	1564 (100)	330 (21)	316 (20)	336 (22)	62 (4)	160 (10)	251 (16)	109 (7)
Gender N (%)								
Female	632 (40)	154 (47)	139 (45)	135 (41)	25 (48)	42 (28)	96 (38)	41 (39)
Male	895 (57)	176 (53)	170 (55)	195 (59)	27 (52)	108 (72)	154 (62)	65 (61)
Age (years) mean/median/range	34/31/5–105	32/31/5–84	35/32/5–85	34/30/5–88	33/27/6–90	30/30/5–85	38/36/10–85	29/26/6–105
Residency N (%)								
Urban	276 (18)	57 (17)	107 (37)	25 (8)	8 (15)	35 (24)	39 (16)	5 (5)
Rural	1219 (78)	271 (83)	186 (64)	294 (92)	44 (85)	113 (76)	209 (84)	102 (95)

Missing values: Gender, *N* = 37; residency, *N* = 69; age, *N* = 142

### Overall results based on case definitions

As per case definition, malaria positivity was found in 17% (268/1564), dengue in 16% (244/1564), scrub typhus in 10% (159/1564), bacteraemia in 8% (124/1564), leptospirosis in 7% (116/1564) and chikungunya in 6% (98/1564). Among malaria cases, 54% (145/268) were *Plasmodium falciparum.* Details of malaria results in this study have been reported previously [7]. Among bacteraemia cases, *Salmonella typhi* or *S. paratyphi* constituted 35% (44/124), *Staphylococcus aureus* 19% (24/124), *E. coli* 9% (11/124) and *Streptococcus pneumoniae* 6% (7/124).

### Centre-wise aetiologies

Table 2 shows prevalence of each aetiology at the different hospitals.

**Table 2:** Aetiology based on standard diagnostic tests grouped by age and study site. *N* = 1564

Diagnose	Total *N* = 1564	5–14 years *N* = 199	15–59 years *N* = 1069	>60 years *N* = 154	Oddanchatram *N* = 330	Ambur *N* = 316	Tezpur *N* = 336	Mungeli *N* = 62	Anantapur *N* = 160	Ratnagiri *N* = 251	Raxaul *N* = 109
Malaria PCR											
Positive	268 (17)	26 (13)	201 (19)	23 (15)	19 (6)	44 (14)	49 (15)	13 (21)	28 (18)	85 (34)	30 (28)
Negative	1144 (73)	151 (76)	773 (72)	126 (82)	299 (91)	230 (73)	244 (73)	39 (63)	96 (60)	160 (64)	76 (70)
Malaria microscopy + PCR											
Positive	66 (4)	4 (2)	57 (5)	4 (3)	3 (1)	7 (2)	14 (4)	2 (3)	9 (6)	29 (12)	2 (2)
Negative	1102 (70)	123 (62)	667 (62)	101 (66)	315 (95)	110 (35)	262 (78)	5 (8)	104 (65)	208 (83)	98 (90)
Bacteraemia											
Positive	124 (8)	13 (7)	102 (10)	4 (3)	31 (9)	9 (3)	36 (11)	0	14 (9)	13 (5)	21 (20)
Negative^a^	1037 (66)	136 (68)	691 (65)	108 (70)	297 (90)	300 (95)	12 (3)	5 (8)	98 (61)	214 (85)	80 (73)
Dengue											
Positive^b^	244 (16)	3 (2)	39 (4)	1 (1)	25 (8)	59 (19)	19 (6)	3 (5)	54 (34)	76 (30)	8 (7)
Negative^c^	1243 (79)	121 (61)	694 (65)	97 (63)	305 (92)	252 (78)	292 (87)	30 (48)	95 (59)	170 (68)	99 (91)
Scrub typhus											
Positive^d^	159 (10)	26 (13)	113 (11)	15 (10)	7 (2)	35 (11)	75 (22)	1 (2)	5 (3)	20 (8)	16 (15)
Negative^e^	1281 (82)	155 (80)	879 (86)	130 (86)	307 (93)	267 (84)	220 (65)	32 (52)	147 (92)	217 (86)	91 (83)
Leptospirosis											
Positive^f^	116 (7)	6 (3)	95 (9)	13 (8)	6 (2)	14 (4)	49 (15)	2 (3)	14 (9)	26 (10)	5 (5)
Negative^g^	1303 (83)	167 (84)	865 (81)	121 (79)	314 (95)	292 (92)	232 (69)	29 (47)	132 (83)	209 (83)	95 (87)
Chikungunya											
Positive	98 (6)	17 (9)	71 (7)	8 (5)	33 (10)	15 (5)	4 (1)	0	29 (18)	13 (5)	4 (4)
Negative	1389 (89)	170 (85)	955 (89)	142 (92)	284 (86)	296 (94)	308 (92)	34 (55)	124 (76)	238 (95)	105 (96)

Data are given as number and percentages among total of patients with case definitions filled, including those with more than one case definition. Discrepancies between positive plus negative results and total number are due to missing values or inconclusive/discrepant test results

^a^Including contaminants (*N* = 40). ^b^Positive MAC ELISA and/or RDT. ^c^Negative MAC ELISA and/or RDT. ^d^Positive IFA on ELISA positives. ^e^ELISA negatives and positive ELISA/IFA negatives. ^f^Positive MAT on ELISA positives. ^g^ELISA negatives and ELISA positive/MAT negatives

The highest prevalence of malaria was found in West-, North- and Central India (Ratnagiri 34%, Raxaul 28% and Mungeli 21%). The highest prevalence of dengue was found in South- and West India (Anantapur 34%, Ambur 19% and Ratnagiri 30%), and a high prevalence of chikungunya (18%) was also found in Anantapur. The highest prevalence of scrub typhus was found in North- and North East India (Tezpur 22% and Raxaul 15%). Tezpur also had the highest prevalence of leptospirosis (15%). Raxaul in North India had as high prevalence as 20% of bacteraemia.

### Overlapping aetiologies

More than one case definition was found in a high number of patients. The overlap between diagnoses is shown in detail in Table 3. The largest overlap was found in chikungunya, where 57% (56/98) had one or more additional case definition and 26% (25/98) overlapped with dengue. Malaria was found in 20% (25/124) among patients with bacteraemia, and among these 48% (12/25) was *P. falciparum*. Among patients with *P. falciparum* and bacteraemia, *Staphylococcus aureus* or Enterobacteriacae including *Salmonella typhi* and *S. paratyphi* were identified. Among patients with positive malaria microscopy confirmed by PCR, 5% (3/66) had bacteraemia.

**Table 3:** Overlap between case definitions (*N* = 1564)

	Tot *N*	Lepto spirosis *N* (%)	Scrub typhus *N* (%)	Dengue *N* (%)	Chikungunya *N* (%)	Bacteraemia *N* (%)	Malaria *N* (%)	Two or more case def. *N* (%)
Leptospirosis	116	–	28 (24)	16 (14)	9 (8)	14 (12)	24 (21)	55 (47)
Scrub typhus	159	28 (18)	–	20 (13)	13 (8)	10 (6)	27 (17)	61 (38)
Dengue	244	16 (7)	20 (8)	–	25 (10)	13 (5)	58 (24)	95 (39)
Chikungunya	98	9 (9)	13 (13)	25 (26)	–	7 (7)	20 (20)	56 (57)
Bacteraemia	124	14 (11)	10 (8)	13 (5)	7 (6)	–	25 (20)	41 (33)
Malaria	268	24 (9)	27 (10)	58 (22)	20 (7)	25 (9)	–	119 (44)
*P. falciparum*	116	11 (9)	17 (15)	33 (28)	8 (7)	12 (10)	–	
Lepto + Scrub	28	–	–	5 (18)	3 (11)	5 (18)	5 (18)	
Lepto + Dengue	16	–	5 (31)	–	1 (6)	3 (19)	4 (25)	
Lepto + Chik	9	–	3 (33)	1 (11)	–	0	1 (11)	
Lepto + Bact	14	–	5 (36)	3 (21)	0	–	4 (29)	
Scrub + Dengue	20	5 (25)	–	–	2 (10)	1 (5)	4 (20)	
Scrub + Chik	13	3 (23)	–	2 (15)	–	0	3 (23)	
Scrub + Bact	10	5 (50)	–	1 (10)	0	–	4 (40)	
Chik + Bact	7	0	0	2	–	–	1	
Bact + Dengue	4	0	0	–	0	–	2	
Lepto + Scrub + Dengue	5	–	–	–	1	1	1	
Scrub + Deng + Mal	4	1	–	–	1	0	–	
Scrub + Bact + Mal	4	2	–	0	0	0		
Dengue + Chik + Mal	2	0	1	–	–	1	–	

Data are given as number of patients and percentages

The association between positive serology (dengue, leptospirosis, scrub typhus and chikungunya) and malaria and septicaemia is shown in Table 4. For each of the four aetiologies, positive serology was equally or more prevalent among malaria positive than negative patients. This was still the case when considering the stricter case definition of clinical malaria, microscopy confirmed by PCR, possibly reducing any bias caused by asymptomatic low parasitaemia. For bacteraemia, the picture was more heterogeneous, where the prevalence of dengue was higher among culture-negative than bacteraemic patients.

**Table 4:** Serology associated with malaria and bacteraemia

Serology	*N*	Malaria PCR (*n* = 1412)			Malaria PCR + microscopy (*N* = 984)			Bacteraemia (*N* = 1161)		
		Positive *N* = 268 *N* (%)	Negative *N* = 1144 *N* (%)	*P*	Positive *N* = 66	Negative *N* = 918	*P*	Positive *N* = 124	Negative^i^ *N* = 1037	*P*
Dengue										
Positive^a^	244	58/258 (22)	170/1118 (15)	0.005	13/65 (20)	120/908 (13)	0.124	13/124 (10)	197/1021 (19)	0.017
Negative^b^	1243	200/258 (78)	948/1118 (85)		52/65 (80)	788/908 (87)		111/124 (90)	824/1021 (81)	
Leptospirosis										
Positive^c^	116	24/248 (10)	85/1061 (8)	0.393	7/60 (12)	72/853 (8)	0.390	14/118 (12)	57/985 (6)	0.011
Negative^d^	1303	224/248 (90)	976/1061 (92)		53/60 (88)	781/853(92)		104/118 (88)	928/985 (94)	
Scrub typhus										
Positive^e^	159	27/250 (11)	119/1078 (11)	0.913	10/63 (16)	104/872 (12)	0.355	10/118 (8)	76/989 (8)	0.762
Negative^f^	1281	223/250 (89)	959/1078 (89)		53/63 (84)	768/872 (88)		108/118 (92)	913/989 (92)	
Chikungunya										
Positive^g^	98	20/261 (8)	70/1117 (6)	0.411	5/64 (8)	57/904 (6)	0.634	7/123 (6)	82/1020 (8)	0.359
Negative^h^	1389	241/261(92)	1047/1117(94)		59/64 (92)	847/904 (94)		116/123(94)	938/1020 (92)	

Data are given as numbers and percentages of tests among total tested with both methods. Discrepancies in numbers are due to missing values. Chi-Square test used for comparison of proportions

^a^Positive MAC ELISA and/or RDT. ^b^Negative MAC ELISA/RDT. ^c^Positive ELISA and MAT. ^d^Negative ELISA and positive ELISA/negative MAT. ^e^Positive ELISA and IFA. ^f^Negative ELISA and positive ELISA/negative IFA. ^g^Positive ELISA. ^h^Negative ELISA ^i^Including 40 samples with contaminants

### Performance of test systems

Table 5 shows the test results of routine diagnostic tests and assesses their performance compared to reference methods.

**Table 5:** Results of tests and performance of routine diagnostic methods compared to reference tests. *N* = 1564

Diagnostic method	*N*	Positive *N* (%)	Negative *N* (%)	Equivocal or discrepancy^a^ *N* (%)	Missing data *N*
Leptospira					
ELISA	1502	201 (14)	1240 (83)	61 (4)	62
MAT on ELISA positives	179	116 (65)	63 (35)		22
MAT on ELISA negatives	52	32 (62)	20 (38)		1188
Scrub typhus					
ELISA	1504	313 (21)	1180 (78)	11 (1)	60
IFA on ELISA positives	260	159 (61)	101 (39)		53
IFA on ELISA negatives	107	12 (11)	95 (89)		1073
Dengue					
NS1/IgM/IgG Combo (RDT)	1465	124 (8)	1318 (90)	23 (2)	99
MAC ELISA	1400	177 (13)	1064 (76)	159 (11)	164
RDT and/or MAC ELISA	1501	244 (16)	1243 (83)	14 (1)	63
MAC ELISA on RDT positives	118	57 (48)	48 (41)	13 (11)	6
MAC ELISA on RDT negatives	1225	102 (8)	989 (81)	134 (11)	93
Chikungunya					
ELISA	1487	98 (7)	1389 (93)		77
Blood culture	1161	164 (14)	997 (86)		403
Pathogenic	1161	124 (11)			
*Neisseria* spp.	124	1 (1)			
*S. aureus*	124	24 (19)			
*Enterococci* spp.	124	2 (2)			
*E. faecalis*	124	1 (1)			
*S. pneumoniae*	124	7 (6)			
*S. pyogenes*	124	1 (1)			
*Streptococci* spp.	124	1 (1)			
*S. typhi/paratyphi*	124	44 (35)			
*Klebsiella* spp	124	1 (1)			
*E. coli*	124	11 (9)			
*Enterobacter spp*	124	1 (1)			
*Acinetobacter*	124	4 (3)			
*Burkholderia cepacia*	124	1 (1)			
*Pseudomonas* spp	124	1 (1)			
*Proteus*	124	2 (2)			
Unidentified^b^	124	22 (18)			
Contaminants^c^	1161	40 (3)			
Malaria					
Genus specific PCR	1412	268 (19)	1144 (81)		152
Species PCR or sequencing	251				17
*P. falciparum*	251	116 (46)			
*P. vivax*	251	96 (38)			
*P. malariae*	251	9 (4)			
*P. falciparum + vivax*	251	27 (11)			
*P. falciparum + malariae*	251	2 (1)			
*P. vivax + malariae*	251	1 (0.4)			
Microscopy	1263	96 (8)	1167 (92)		301
Microscopy and genus PCR	1168	66 (6)	918 (79)	184 (16)	396
Microscopy, genus PCR and RDT	1163	41 (4)	906 (78)	216 (19)	401

^a^Discrepancy between tests performed at study sites and reference laboratory, or equivocal results

^b^Cocci (*N* = 5), gram negative (*N* = 4), gram positive (*N* = 7), unidentified (*N* = 6)

^c^ *Bacillus* (*N* = 14), coagulase negative staphylococci (*N* = 7), *Corynebacterium* (*N* = 4), diphteroids (*N* = 1), micrococci (*N* = 10), undefined contaminants (*N* = 4)

Using IFA as gold standard, positive predictive value for Scrub typhus IgM ELISA was 61% (159/260), and negative predictive value was 89% (95/107).

Compared to MAT, positive predictive value for *Leptospira* IgM ELISA was 65% (116/179) while negative predictive value was as low as 38% (20/52). The study was not designed to evaluate sensitivity and specificity, as gold standard tests were not performed on all ELISA negatives.

Using malaria PCR as gold standard, the sensitivity of routine microscopy was 29% (66/228) and RDT 24% (65/268), as reported previously [7].

Sensitivity and specificity for dengue tests were not calculated because gold standard was positive RDT and/or MAC ELISA, and the two tests are expected to be positive during different intervals of the illness. Indeed, only 46% (57/124) of RDT positives were positive by MAC ELISA, probably reflecting early infections detected only by NS1Ag.

## Discussion

This study of aetiology of undifferentiated fever in rural India using standard diagnostic tests, revealed a high prevalence of malaria and dengue. However, there was a strikingly high prevalence of overlap of case definitions. An overlap with one or more other case-definition was found for all diagnosed diseases, ranging from 33% (bacteraemia) to 57% (chikungunya) (Table 3). The highest frequency of overlap was found in chikungunya where dengue was simultaneously diagnosed in 26% (25/98), followed by leptospirosis, where scrub typhus was found in 24% (28/116).

Cross reactivity, or background positivity due to previous infections, are well known limitations of serological tests, and fourfold rise of titer in convalescence samples or a high acute phase titer is recommended to confirm a diagnosis. Convalescent samples were not available in this study, reflecting a real life situation in resource poor settings where tests for follow up in recovered patients are usually not collected.

Detecting a pathogen directly by PCR or culture is more specific than indirect diagnosis by antibody detection, and the diagnoses of malaria and bacteraemia are therefore likely to be more specific than leptospirosis, scrub typhus, chikungunya and dengue in this study. Positive serological tests for dengue, leptospirosis, scrub typhus and chikungunya were common also in patients with malaria and bacteraemia (Table 4), suggesting low specificity of the serological tests. Although coinfections are possible, it is more likely that multiple fulfilled case definitions in a high proportion of patients are due to cross reactivity and background positivity, reflecting that the diseases detected by serology are endemic in the area, rather than high prevalence of coinfections. The findings in the present study emphasises the importance of interpreting diagnostic tests in a clinical context together with symptoms, clinical findings and biochemical tests.

### Malaria

Malaria parasites were detected by PCR in 17% (268/1564) among patients included, and among these 54% (145/268) were *P. falciparum*, as reported previously [7]. Due to high sensitivity of malaria PCR compared to microscopy and RDT, some PCR positive cases may potentially have had asymptomatic low parasitemia controlled by immunity, or recently been treated for malaria, and their fever caused by another infection [7, 9]. As reported previously, microscopy had low sensitivity (29%, 66/228) but high specificity (98%, 918/940) compared to PCR, and a very strict case definition of clinical malaria as cause of acute fever can be defined as a positive microscopy confirmed by PCR [7]. The prevalence of malaria by microscopy confirmed by PCR was 6% (66/1168).

### Bacteraemia

Blood stream infection with pathogenic bacteria was diagnosed in 8% (124/1564), and among these *Salmonella typhi* or *S. paratyphi* were found in 35% (44/124), reflecting the high prevalence of enteric fever in India. Enteric fever is closely associated with poor sanitation, lack of safe water supply and treatment failures due to antimicrobial resistance and is still reported as the most common blood stream infection in India and in South Asia [10–13]. The second most common microbe identified was *S. aureus* (19% 24/124), followed by *E.-coli* (9%, 11/124) and *S. pneumoniae* (6%, 7/124).

### Dengue

Dengue and severe dengue because of immune enhancement due to a previous infection with another serotype is an increasing problem in India [14, 15]. India is estimated to contribute 34% (33/96 million) of the total global burden of dengue [16], with increasing incidence both of dengue and outbreaks of severe dengue [17, 18]. The risk of severe dengue is high, as more than 25% of the population in Delhi has been reported to have had a past infection [17, 19]. In line with the high prevalence reported in previous studies, dengue was found in as much as 16% (244/1564) in the present study, highest in the sites in South- and West India.

Rapid tests combining detection of non-structural protein 1 (NS1) antigen and IgM/IgG are used in routine diagnostics, as they have high sensitivity both during the viremic early phase of infection when NS1 is produced and after more than five days when IgM can be detected [20]. IgM capture ELISA (MAC ELISA) is used as reference method, but is less sensitive than NS1Ag until day five of infection. Case definition used in the present study was therefore a positive test with RDT and/or ELISA, in order not to miss out early infections detected by NS1Ag. However, background positivity is a potential limitation since MAC ELISA can be positive for several months after infection [21]. Although NS1 antigen is less prone to give cross reactivity than IgM antibodies, combination tests have shown some false positive reactions in non-dengue infections, most commonly in chikungunya [20, 21].

### Chikungunya

A large outbreak of *Chikungunya* was reported in Ahmedabad in India in 2006 [22]. Sharing the same vector, chikungunya is likely to occur during dengue outbreaks, and in a study during a dengue outbreak in Delhi in 2010, 10% (66/666) positive chikungunya cases were diagnosed among dengue IgM negative fever patients [23]. Sporadic outbreaks of chikungunya has been reported in India since 1963, in 2006 affecting 13 states with 1.4 million suspected cases [23], with high numbers in Andhra Pradesh, Tamil Nadu and Maharashtra. This supports the finding in the present study of highest prevalence of chikungunya in Anantapur (Andhra Pradesh), Oddanchatram and Ambur (Tamil Nadu) and Ratnagiri (Maharashtra).

### Leptospirosis

Leptospirosis is transmitted by urine from infected animals (rats, cattle, pigs) and is endemic particularly in the Andaman and Nicobar group of islands (“Andaman haemorrhagic fever”) [24]. In AUF studies from South- and Northern India, leptospirosis was reported in 3% and 0.1% respectively [11, 12]. In the present study leptospirosis was found in 7% (116/1564), and cases were identified at all study sites.

Culturing *Leptospira* is unreliable, and the gold standard is therefore serology confirmed by MAT. MAT detects IgM and IgG antibodies against a pool of live antigens from different *Leptospira* serovars. A MAT titer >100 is considered positive, but a fourfold rise in convalescence titer or a high single acute phase titer (>200–1600 depending on endemicity) supports the diagnosis [25]. Following acute leptospirosis, both IgM ELISA and MAT remain positive for several years after infection, with duration differing between serogroups [26]. In one prospective study from Barbados, positive MAT was found up to 11 years after infection, with highest prevalence after serogroup Autumnalis infection where 20% had MAT titer >800 after four years [26]. In the present study Autumnalis was found in 7%. Leptospira serovar prevalence and distribution in this study has been reported previously [27]. *Leptospira* IgM ELISA has been reported positive in 40% and 5% one and six years after infection respectively [26]. Discrimination between acute and previous infection in the present study is limited by lack of convalescent samples, and a low MAT cut-off titer of 100. However, high prevalence of antibodies in all study sites suggests that the disease is endemic in the areas.

### Scrub typhus

Scrub typhus is transmitted by mites who live on rats. The disease is, similar to leptospirosis, associated with agricultural work and rural dwelling [28]. Two studies have reported prevalence of 14% and 47% among hospitalized febrile patients in North- and South India respectively [11, 12]. The disease is endemic in various parts of India, but underreported [1, 29–35]. In the present study, scrub typhus was found in 10% (159/1564), and the disease was identified at all study sites.

Serology confirmed by IFA, ideally confirmed by rise of titer in convalescent samples and/or by cut-off values based on endemicity, remains the mainstay of diagnostics since isolation of the bacteria is not possible and PCR from blood has low sensitivity [36]. Sensitivity of IFA may be influenced by antigen variation. Usually antigens from three serotypes (Karp, Kato and Gilliam) are used, while additional antigens may be present in different areas [30, 36]. In the present study, discrimination between previous scrub typhus and acute infection is limited by the lack of convalescent samples. Also an optic density (OD) value of 0.5 may be in the lower range and, thereby, in some cases reflect background positivity.

### Potential coinfections

Although background positivity or cross reactivity in serology, and potential subclinical infections in malaria, may have given positive test results in some cases, some of the overlapping aetiologies have probably been due to true clinically relevant coinfections.

Coinfections could occur principally by two different mechanisms; by contracting multiple infections at the same time, or increased pathogenicity of a simultaneous subclinical infection due to immune reactions.

The risk of bacterial sepsis is increased in severe malaria, through immune mediated barrier dysfunction in the gut and bacterial translocation, as well as IL-10 mediated decreased control of bacteraemia [37, 38]. In clinical studies, invasive infection, frequently with *Salmonella* spp. or other Gram-negatives, are found both in *P. falciparum* and *P. vivax* malaria [39, 40], which supports the finding of as much as 9% (25/268) bacteraemia among malaria patients in the present study. On the other hand, asymptomatic malaria controlled by immunity may obscure correct diagnosis of bacterial sepsis [41–43], and an undefined proportion of the malaria positive patients among those with bacteraemia may have had subclinical malaria in the present study.

In a study among Thai rice farmers with leptospirosis diagnosed with 4-fold rise in titer or a single high titer, as many as nine among 22 patients had coinfection with scrub typhus confirmed by serology and eschar or clinical characteristics [44]. Although a very high overlap between positive tests for scrub typhus and leptospirosis in the present study suggests background positivity or cross reactivity, a proportion of the patients may have had coinfections taking into consideration the similar exposure risk.

True coinfections with malaria and scrub typhus, diagnosed by clinical characteristics and eschar or PCR, have also been reported in India [45–47]. However, the high level of positive scrub typhus serology in single samples found in other Indian studies [12, 48], raises the same question as in the present study where 10% (27/268) of malaria cases had positive scrub typhus serology, do the results reflect true coinfections, or cross reactivity or background positivity?

In the mosquito borne infections dengue, malaria and chikungunya, outbreaks occur during rainy seasons and although the specific vector is different for malaria, coinfections are not unlikely. This was shown in a study from India during a dengue outbreak, where 7% (27/367) of dengue cases had coinfection with malaria [49]. As much as 22% (58/268) of malaria cases had positive dengue tests in the present study (Tables 3 and 4).

A high level of coinfections with dengue and chikungunya was shown during a dengue outbreak in Delhi in 2006 using PCR as the method for detection. Among 17 chikungunya positive patients, six were co-infected with dengue virus [50]. Ten percent coinfection was found in a study from Mumbai [51]. Dengue and chikungunya virus share a common mosquito vector, the daytime biting *Aedes aegypti* and *A. albopictus*, and are present in similar geographical regions. In the present study, dengue and chikungunya both had high prevalence in Anantapur, supporting the notion that coinfections as well as cross reactivity could explain some overlap between dengue and chikungunya.

## Conclusion

A high prevalence of malaria and dengue, and a high overlap between case definitions were found in this study. The overlap probably reflects an undefined level of previous infections, cross reactivity and subclinical infections in the population, rather than high prevalence of coinfections. These limitations of routine diagnostic tests should be taken into consideration when approaching the patient with acute undifferentiated fever in India.

## Abbreviations

AUF: Acute Undifferentiated Fever
CMC: Christian Medical College
ELISA: Enzyme-linked immunosorbent assay
IFA: Immunofluorescence assay
IL-10: Interleukin 10
MAC-ELISA: IgM Antibody Capture ELISA
MAT: Microscopic Agglutination Test
NIV: National Institute of Virology
NS1: Non-structural protein 1
OD: Optical Density
PCR: Polymerase Chain Reaction
RDT: Rapid Diagnostic Test

## References

[1] John TJ, Dandona L, Sharma VP, Kakkar M (2011). Continuing challenge of infectious diseases in India. Lancet.

[2] Kumar A, Valecha N, Jain T, Dash AP (2007). Burden of malaria in India: retrospective and prospective view. The American journal of tropical medicine and hygiene.

[3] Dhingra N, Jha P, Sharma VP, Cohen AA, Jotkar RM, Rodriguez PS, Bassani DG, Suraweera W, Laxminarayan R, Peto R (2010). Adult and child malaria mortality in India: a nationally representative mortality survey. Lancet.

[4] WHO: World malaria report 2014. 2014.

[5] Mathai D, Kumar VA, Paul B, Sugumar M, John KR, Manoharan A, Kesavan LM (2015). Fecal carriage rates of extended-spectrum beta-lactamase-producing Escherichia coli among antibiotic naive healthy human volunteers. Microb Drug Resist.

[6] D'Acremont V, Kilowoko M, Kyungu E, Philipina S, Sangu W, Kahama-Maro J, Lengeler C, Cherpillod P, Kaiser L, Genton B (2014). Beyond malaria--causes of fever in outpatient Tanzanian children. N Engl J Med.

[7] Haanshuus CG, Chandy S, Manoharan A, Vivek R, Mathai D, Xena D, Singh A, Langeland N, Blomberg B, Vasanthan G (2016). A High Malaria Prevalence Identified by PCR among Patients with Acute Undifferentiated Fever in India. PLoS One.

[8] Haanshuus CG, Mohn SC, Morch K, Langeland N, Blomberg B, Hanevik K (2013). A novel, single-amplification PCR targeting mitochondrial genome highly sensitive and specific in diagnosing malaria among returned travellers in Bergen, Norway. Malar J.

[9] Bousema T, Okell L, Felger I, Drakeley C (2014). Asymptomatic malaria infections: detectability, transmissibility and public health relevance. Nature reviews Microbiology.

[10] Wain J, Hendriksen RS, Mikoleit ML, Keddy KH, Ochiai RL (2015). Typhoid fever. Lancet.

[11] Chrispal A, Boorugu H, Gopinath KG, Chandy S, Prakash JA, Thomas EM, Abraham AM, Abraham OC, Thomas K (2010). Acute undifferentiated febrile illness in adult hospitalized patients: the disease spectrum and diagnostic predictors - an experience from a tertiary care hospital in South India. Trop Dr.

[12] Mittal G, Ahmad S, Agarwal RK, Dhar M, Mittal M, Sharma S (2015). Aetiologies of Acute Undifferentiated Febrile illness in Adult Patients - an Experience from a Tertiary Care Hospital in Northern India. Journal of clinical and diagnostic research : JCDR.

[13] Kanungo S, Dutta S, Sur D (2008). Epidemiology of typhoid and paratyphoid fever in India. Journal of infection in developing countries.

[14] Guzman MG, Harris E (2015). Dengue. Lancet.

[15] Organization. WH (2009). Dengue: Guidelines for Diagnosis, Treatment, Prevention and control: New Edition.

[16] Bhatt S, Gething PW, Brady OJ, Messina JP, Farlow AW, Moyes CL, Drake JM, Brownstein JS, Hoen AG, Sankoh O (2013). The global distribution and burden of dengue. Nature.

[17] Siddiqui O, Chakravarti A, Abhishek KS (2016). Dengue: Lessons of an Outbreak. Journal of clinical and diagnostic research : JCDR.

[18] Dar L, Broor S, Sengupta S, Xess I, Seth P (1999). The first major outbreak of dengue hemorrhagic fever in Delhi, India. Emerg Infect Dis.

[19] Vikram K, Nagpal BN, Pande V, Srivastava A, Saxena R, Anvikar A, Das A, Singh H, Anushrita GSK (2016). An epidemiological study of dengue in Delhi, India. Acta Trop.

[20] Blacksell SD, Jarman RG, Bailey MS, Tanganuchitcharnchai A, Jenjaroen K, Gibbons RV, Paris DH, Premaratna R, de Silva HJ, Lalloo DG (2011). Evaluation of six commercial point-of-care tests for diagnosis of acute dengue infections: the need for combining NS1 antigen and IgM/IgG antibody detection to achieve acceptable levels of accuracy. Clinical and vaccine immunology : CVI.

[21] Mardekian SK, Roberts AL (2015). Diagnostic Options and Challenges for Dengue and Chikungunya Viruses. Biomed Res Int.

[22] Mavalankar D, Shastri P, Bandyopadhyay T, Parmar J, Ramani KV (2008). Increased mortality rate associated with chikungunya epidemic, Ahmedabad, India. Emerg Infect Dis.

[23] Ramachandran VG, Das S, Roy P, Hada V, Mogha NS (2016). Chikungunya: a reemerging infection spreading during 2010 dengue fever outbreak in National Capital Region of India. Virusdisease.

[24] Budihal SV, Perwez K (2014). Leptospirosis diagnosis: competancy of various laboratory tests. Journal of clinical and diagnostic research : JCDR.

[25] Cumberland P, Everard CO, Levett PN (1999). Assessment of the efficacy of an IgM-elisa and microscopic agglutination test (MAT) in the diagnosis of acute leptospirosis. The American journal of tropical medicine and hygiene.

[26] Cumberland P, Everard CO, Wheeler JG, Levett PN (2001). Persistence of anti-leptospiral IgM, IgG and agglutinating antibodies in patients presenting with acute febrile illness in Barbados 1979-1989. Eur J Epidemiol.

[27] Chandy S, Kirubanandhan L, Hemavathy P, Khadeeja AM, Kurian SJ, Venkataraman K, Morch K, Mathai D, Manoharan A (2017). Serovar prevalence of Leptospira in semirural India and the development of an IgM-based indirect ELISA. Journal of infection in developing countries.

[28] Varghese GM, Trowbridge P, Janardhanan J, Thomas K, Peter JV, Mathews P, Abraham OC, Kavitha ML. Clinical profile and improving mortality trend of scrub typhus in South India. International journal of infectious diseases: IJID: official publication of the International Society for Infectious Diseases. 2014. 10.1016/j.ijid.2014.02.009 24661931

[29] Kumar V, Kumar V, Yadav AK, Iyengar S, Bhalla A, Sharma N, Aggarwal R, Jain S, Jha V (2014). Scrub typhus is an under-recognized cause of acute febrile illness with acute kidney injury in India. PLoS Negl Trop Dis.

[30] Varghese GM, Janardhanan J, Mahajan SK, Tariang D, Trowbridge P, Prakash JA, David T, Sathendra S, Abraham OC (2015). Molecular epidemiology and genetic diversity of Orientia tsutsugamushi from patients with scrub typhus in 3 regions of India. Emerg Infect Dis.

[31] Mahajan SK, Rolain JM, Kashyap R, Bakshi D, Sharma V, Prasher BS, Pal LS, Raoult D (2006). Scrub typhus in Himalayas. Emerg Infect Dis.

[32] Viswanathan S, Muthu V, Iqbal N, Remalayam B, George T (2013). Scrub typhus meningitis in South India--a retrospective study. PLoS One.

[33] Khan SA, Dutta P, Khan AM, Topno R, Borah J, Chowdhury P, Mahanta J (2012). Re-emergence of scrub typhus in northeast India. International journal of infectious diseases : IJID : official publication of the International Society for Infectious Diseases.

[34] Isaac R, Varghese GM, Mathai E, J M, Joseph I (2004). Scrub typhus: prevalence and diagnostic issues in rural Southern India. Clinical infectious diseases : an official publication of the Infectious Diseases Society of America.

[35] Dass R, Deka NM, Duwarah SG, Barman H, Hoque R, Mili D, Barthakur D (2011). Characteristics of pediatric scrub typhus during an outbreak in the North Eastern region of India: peculiarities in clinical presentation, laboratory findings and complications. Indian J Pediatr.

[36] Koh GC, Maude RJ, Paris DH, Newton PN, Blacksell SD (2010). Diagnosis of scrub typhus. The American journal of tropical medicine and hygiene.

[37] Chau JY, Tiffany CM, Nimishakavi S, Lawrence JA, Pakpour N, Mooney JP, Lokken KL, Caughey GH, Tsolis RM, Luckhart S (2013). Malaria-associated L-arginine deficiency induces mast cell-associated disruption to intestinal barrier defenses against nontyphoidal Salmonella bacteremia. Infect Immun.

[38] Lokken KL, Mooney JP, Butler BP, Xavier MN, Chau JY, Schaltenberg N, Begum RH, Muller W, Luckhart S, Tsolis RM (2014). Malaria parasite infection compromises control of concurrent systemic non-typhoidal Salmonella infection via IL-10-mediated alteration of myeloid cell function. PLoS Pathog.

[39] Church J, Maitland K (2014). Invasive bacterial co-infection in African children with Plasmodium falciparum malaria: a systematic review. BMC Med.

[40] Bhattacharya SK, Sur D, Dutta S, Kanungo S, Ochiai RL, Kim DR, Anstey NM, von Seidlein L, Deen J (2013). Vivax malaria and bacteraemia: a prospective study in Kolkata, India. Malar J.

[41] Ganguly S, Saha P, Guha SK, Biswas A, Das S, Kundu PK, Maji AK (2013). High prevalence of asymptomatic malaria in a tribal population in eastern India. J Clin Microbiol.

[42] Hamer DH, Singh MP, Wylie BJ, Yeboah-Antwi K, Tuchman J, Desai M, Udhayakumar V, Gupta P, Brooks MI, Shukla MM (2009). Burden of malaria in pregnancy in Jharkhand State, India. Malar J.

[43] Singh N, Singh MP, Wylie BJ, Hussain M, Kojo YA, Shekhar C, Sabin L, Desai M, Udhayakumar V, Hamer DH (2012). Malaria prevalence among pregnant women in two districts with differing endemicity in Chhattisgarh, India. Malar J.

[44] Watt G, Jongsakul K, Suttinont C (2003). Possible scrub typhus coinfections in Thai agricultural workers hospitalized with leptospirosis. The American journal of tropical medicine and hygiene.

[45] McGready R, Ashley EA, Wuthiekanun V, Tan SO, Pimanpanarak M, Viladpai-Nguen SJ, Jesadapanpong W, Blacksell SD, Peacock SJ, Paris DH (2010). Arthropod borne disease: the leading cause of fever in pregnancy on the Thai-Burmese border. PLoS Negl Trop Dis.

[46] Mahajan SK, Kaushik M, Raina R, Thakur P (2014). Scrub typhus and malaria co-infection causing severe sepsis. Trop Dr.

[47] Kumar S, Kumar PS, Kaur G, Bhalla A, Sharma N, Varma S (2014). Rare concurrent infection with scrub typhus, dengue and malaria in a young female. Journal of vector borne diseases.

[48] Ahmad S, Dhar M, Mittal G, Bhat NK, Shirazi N, Kalra V, Sati HC, Gupta V. A comparative hospital-based observational study of mono- and co-infections of malaria, dengue virus and scrub typhus causing acute undifferentiated fever. European journal of clinical microbiology & infectious diseases : official publication of the European Society of Clinical Microbiology. 2016; 10.1007/s10096-016-2590-3 26851948

[49] Mohapatra MK, Patra P, Agrawala R (2012). Manifestation and outcome of concurrent malaria and dengue infection. Journal of vector borne diseases.

[50] Chahar HS, Bharaj P, Dar L, Guleria R, Kabra SK, Broor S (2009). Co-infections with chikungunya virus and dengue virus in Delhi, India. Emerg Infect Dis.

[51] Londhey V, Agrawal S, Vaidya N, Kini S, Shastri JS, Sunil S (2016). Dengue and Chikungunya Virus Co-infections: The Inside Story. J Assoc Physicians India.

